# Applications of Flexible Ultrasonic Transducer Array for Defect Detection at 150 °C

**DOI:** 10.3390/s130100975

**Published:** 2013-01-15

**Authors:** Jeanne-Louise Shih, Kuo-Ting Wu, Cheng-Kuei Jen, Chun-Hsiung Chiu, Jing-Chi Tzeng, Jiunn-Woei Liaw

**Affiliations:** 1 Department of Electrical and Computer Engineering, McGill University, 3480 University Street, Montreal, QC H3A 2K6, Canada; E-Mails: shih.jeanne@gmail.com (J.-L.S.); chengkuei.jen@gmail.com (C.-K.J.); 2 National Research Council Canada, Boucherville, QC J4B 6Y4, Canada; E-Mail: kuo-ting.wu@cnrc-nrc.gc.ca; 3 Department of Mechanical Engineering, Chang Gung University, 259 Wen-Hwa 1st Rd., Kwei-Shan, Tao-Yuan 333, Taiwan; E-Mails: cch741018@gmail.com (C.-H.C.); f16265321@hotmail.com (J.-C.T.); 4 Center for Biomedical Engineering, Chang Gung University, 259 Wen-Hwa 1st Rd., Kwei-Shan, Tao-Yuan 333, Taiwan; 5 Healthy Aging Research Center, Chang Gung University, 259 Wen-Hwa 1st Rd., Kwei-Shan, Tao-Yuan 333, Taiwan

**Keywords:** flexible ultrasonic transducer array, PZT sol-gel, pulse-echo mode, pitch-catch mode, total focusing method, phased-array imaging, defect detection

## Abstract

In this study, the feasibility of using a one dimensional 16-element flexible ultrasonic transducer (FUT) array for nondestructive testing at 150 °C is demonstrated. The FUT arrays were made by a sol-gel sprayed piezoelectric film technology; a PZT composite film was sprayed on a titanium foil of 75 μm thickness. Since the FUT array is flexible, it was attached to a steel pipe with an outer diameter of 89 mm and a wall thickness of 6.5 mm at 150 °C. Using the ultrasonic pulse-echo mode, pipe thickness measurements could be performed. Moreover, using the ultrasonic pulse-echo and pitch-catch modes of each element of FUT array, the defect detection was performed on an Al alloy block of 30 mm thickness with a side-drilled hole (SDH) of ϕ3 mm at 150 °C. In addition, a post-processing algorithm based on the total focusing method was used to process the full matrix of these A-scan signals of each single transmitter and multi-receivers, and then the phase-array image was obtained to indicate this defect- SDH. Both results show the capability of FUT array being operated at 150 °C for the corrosion and defect detections.

## 1. Introduction

Multiple ultrasonic transducer (UT) arrays [1–4], e.g., phase-array UT, are becoming attractive for the applications of nondestructive testing (NDT) and structural health monitoring (SHM) due to the efficient inspection, compared to a single element UT. The main purposes of ultrasonic measurement are for the defects and corrosions detections in materials. Moreover, these ultrasonic measurements are often required to operate under high temperatures condition [1,2,4] for the in-service inspections of electrical power plants and petrochemical plants *etc.* The crucial challenge of higher-temperature condition is that it could induce the damage of transducer due to the internal disbonding caused by the thermal expansion. Therefore, special high-temperature UTs and special couplant should be developed and employed to overcome the high temperature conditions. In addition, the requirements for these measurements performed on curved surfaces, e.g., pipes and reactors, are usually encountered. Therefore, to develop flexible UT arrays [5] applicable at high temperature [4] and on curved surface is extremely of importance to the industrial in-service inspection.

In this study, the feasibility of using one dimensional (1D) flexible ultrasonic transducer (FUT) arrays, made by a technology of PZT sol-gel [6–8] sprayed piezoelectric thick film on Ti foil [9,10], for defect detection at 150 °C is demonstrated. First, the ultrasonic thickness measurement of a straight steel pipe using the home-made FUT array attached on the outer surface of pipe is performed at 150 °C for the application of corrosion detection, where the pulse-echo signals are used. Since the internal and external corrosions can cause gradual decay and deterioration of pipes to reduce the strength by thinning the wall thickness, the corrosion detection using thickness measurement at high temperature is necessary to ensure the safety and security of structures. Moreover, the ultrasonic defect detection using FUT array on an Al alloy block with a side-drilled hole (SDH) at 150 °C is also demonstrated. In addition, the total focusing method (TFM) is used to process the pulse-echo and pitch-catch signals of FUT array for synthesizing the phased-array image [11–13]. Since the in-service NDT and SHM under high temperature conditions are needed for industry, especially for petrochemical and nuclear power plants, the FUT arrays may show their applicability for the corrosion and defect detections.

## 2. Fabrication of Piezoelectric 1D FUT Array

**Figure 1.**
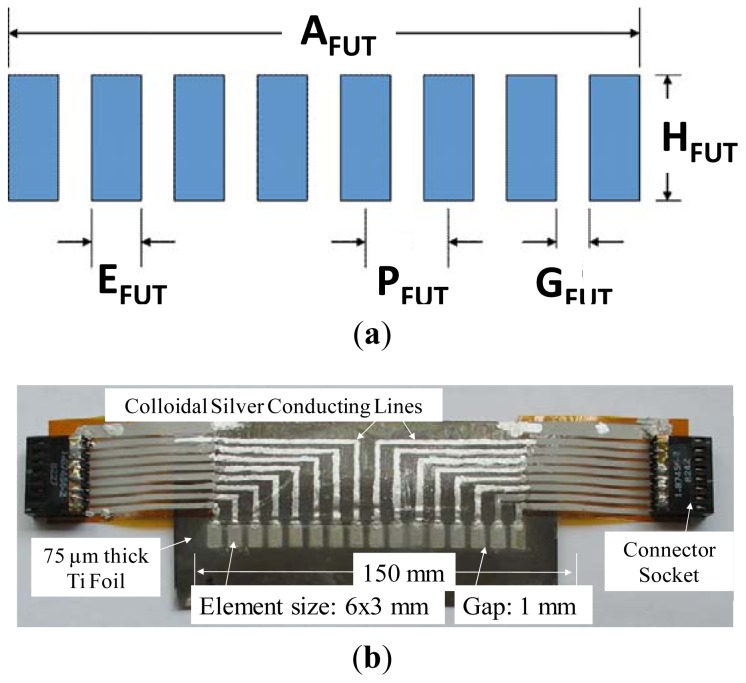

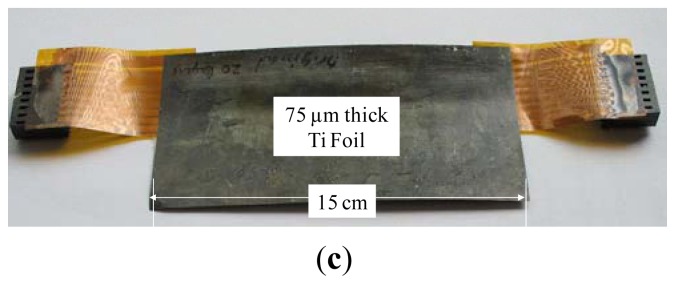
(**a**) Configuration of FUT array: aperture (A_FUT_), element size (E_FUT_), height (H_FUT_), gap (G_FUT_) and pitch (P_FUT_). (**b**) Top and (**c**) bottom view of a 1D 16-element FUT array on a 75 μm thick Ti foil.

The configuration of FUT array is shown in Figure 1(a), where aperture (A_FUT_), element size (E_FUT_), height (H_FUT_), gap (G_FUT_) and pitch (P_FUT_). The top and bottom views of a typical FUT array on a Ti foil are shown in Figure 1(b,c), respectively, where each element size of the FUT array is 6 mm × 3 mm and the gap between two adjacent elements is 1 mm (*i.e.*, 4 mm pitch) on 75 μm thick Ti foil. The fabrication of the FUT array consists of six main steps [7,9]: (1) preparation of a high dielectric constant lead-zirconate-titanate (PZT) solution [8], (2) ball milling of the piezoelectric PZT powders in a PZT solution to submicron sizes, (3) sensor spraying using slurries to produce a film with thickness of 5 to 20 μm, (4) heat treatment to produce a solid PZT composite (PZT-c) thick film, (5) Corona poling to obtain piezoelectricity, and (6) colloidal silver spraying with a mask to deposit the top electrodes and electrically conducting lines. Steps (3,4) are performed multiple times to produce an optimal PZT-c film thickness for the specified ultrasonic operating frequency (center frequency). Normally, the thicker the PZT-c film, the lower the center frequency. If the final thickness of the PZT-c film is about 83 μm, the center frequency of each element is about 6 to 7 MHz. An insulation layer is coated onto the Ti substrate (foil) under the conducting line stripes. All the components shown in Figure 1(b) may sustain and have been tested at up to 150 °C. Each of the two connector sockets is connected to eight FUT array elements and one electrical ground line which is the Ti substrate. We also fabricated a 1D 16-element array on 75 μm thick SS foils, and compared the performances of Ti foil with SS foil. It is noted that the signal strength of the FUTs made on Ti foil is normally a few dB better than that achieved from FUTs made on SS foil because of a decrease in substrate oxidation during heat treatment. However, FUTs using SS foils may be brazed onto steel substrates such as pipes for SHM purposes at HT [9]. Finally another insulation layer acting as a protection layer to prevent moisture, which may also sustain temperatures of up to 150 °C was coated, but not shown in Figure 1.

For the defect detection, another FUT array (element size: 10 mm × 2 mm, gap: 1 mm) on a Ti foil of 75 μm was also produced. In addition, the other smaller FUT array (element size: 9 mm × 2mm, gap: 0.5 mm) on a thinner Ti foil of 35 μm was also fabricated. The thicknesses of both two PZT-c films are about 80 μm to tune the center frequency of each element within 7 and 8 MHz.

## 3. Theory

**Figure 2.**
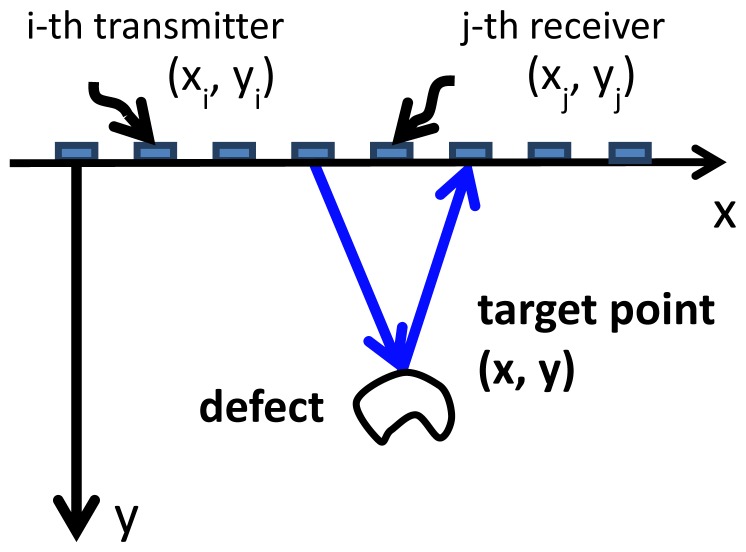
Schematic of TFM for pitch-catch signals of the *i*th transmitter and the *j*th receiver.

Using the FUT array, the pitch-catch modes of the *i*th transmitter and the *j*th receiver can be performed for all elements to acquire the A-scan signal *A_ij_*(*t*), as shown in Figure 2. After storage of the full matrix of *A_ij_*(*t*), a post-processing algorithm can be applied to reconstruct the phased-array imaging. Based on the TFM [11,12] or the so-called sampling phased array algorithm [13], the synthesized signal *S_j_* (**x**, *t*) captured by the *j*^th^ receiver, which scatters from a target point **x** as waves generated by all transmitters with different time delays simultaneously arrive at the target point, is reconstructed as:

(1) $$S_{j}(x,t)=\sum_{i}A_{ij}(t-t_{ij})$$

where *t_ij_* is time shift, *t_ij_* = *d_ij_/V*, and *d_ij_* is the distance, *d_ij_* = |**x** – **x***_i_*| + |**x** – **x***_j_*|. Here v is the longitudinal wave speed in medium, and **x***i* is the position vector of the *i*th element. We assume *Sj* (**x**, *t*) is the real part of an analytic signal. Therefore using the Hilbert transform, we can obtain the envelope of the analytic signal, and find the peak value for the *jt*h receiver. Subsequently, the intensity of the response of multi-transmitters with multi-receivers for the target point **x** is calculated by combining these peak values of all receivers. Sweeping the inspected area, the phased-array imaging can be obtained to indicate the location of defect and bottom. Since the performance of each element is not the same, the calibration and normalization of these signals are necessary prior to the calculation of phased-array imaging.

## 4. Experimental Results and Discussion

**Figure 3.**
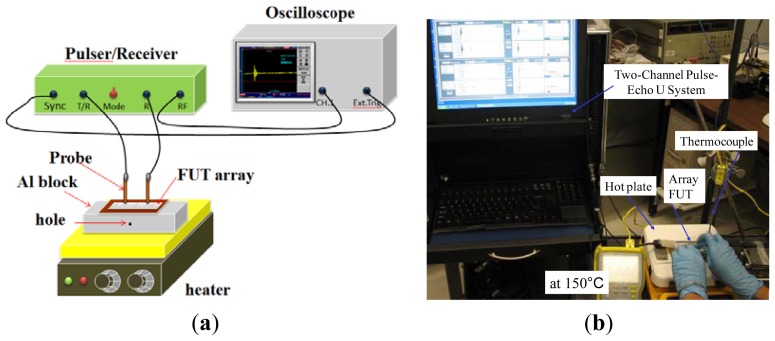
(**a**) Schematic and (**b**) experimental setup for ultrasonic measurements of FUT array on a metal block at 150 °C.

The schematic of FUT array for ultrasonic measurement is shown in Figure 3(a). Figure 3(b) is the photo of our experimental setup for the ultrasonic measurements carried out at 150 °C using an ultrasonic pulser-receiver system for the 1D 16-element FUT array 6 mm × 3 mm and the gap between them is 1 mm (*i.e.*, 4 mm pitch) on 75 μm thick Ti foil, as shown in Figure 1(b).

**Figure 4.**
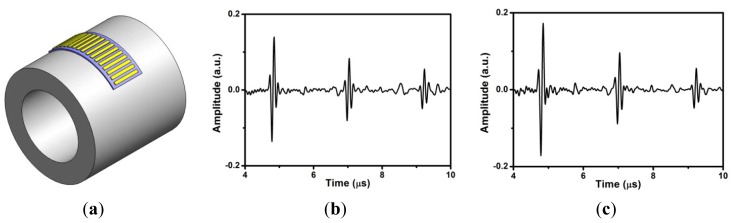
(**a**) Ultrasonic pulse-echo measurements of FUT array (element size: 6 mm × 3 mm, gap: 1 mm) for a steel pipe of OD: 89 mm with 6.5 mm thickness at 150 °C, where (**b**) is the signal of element 5 and (**c**) the signal of element 9.

Since the FUT array is flexible, it was pressed and then attached to a steel pipe along the radial direction for ultrasonic thickness measurements at 150 °C as shown in Figure 4(a), where a high-temperature couplant was added in between the FUT array and pipe. The couplant is a 5W-30 car engine oil lubricant.

The outer diameter (OD) of the pipe is 89 mm, and the wall thickness 6.5 mm. Figure 4(b,c) show the simultaneous pulse-echo measurements for the elements 5 and 9 with the same data recording settings, where only the 1st, 2nd, and 3rd echoes are plotted. These multiple echoes result from the reflection from the inner surface of the pipe. The center frequencies for the 1st echo of element 5 and element 9 are 7 and 6 MHz, respectively. Normally, these ultrasonic signals of FUT are broadband. These results at 150 °C are about 7 dB weaker than those measured at room temperature. The average time difference between two adjacent echoes is about 2.18 μs. Since the longitudinal wave speed in steel is about 5,910 m/s, the estimated thickness is about 6.442 mm. It was demonstrated that the thickness measurement of FUT array at high temperature can be applied to the in-service inspection of the internal corrosion of pipe, particularly the erosion corrosion inside the elbow.

**Figure 5.**
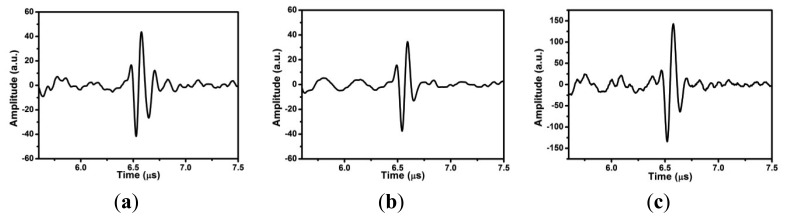
(**a**) The pulse-echo signal *A*_44_, (**b**) the pitch-catch signal *A*_45_, and (**c**) the synthesized signal *S*_4_ of the target point (10 mm, 13.5 mm) for 8-elements FUT array (element size: 10 mm × 2 mm, gap: 1 mm) inspecting an Al alloy block with a SDH of ϕ3 mm at the middle at 150 °C.

Furthermore, in order to demonstrate the feasibility of the 1D FUT array as a phase array transducer operable at 150 °C, another 16-element FUT array with an element dimension of 10 mm × 2 mm and a gap of 1 mm on a Ti foil of 75 μm was used for defect detection. The TFM algorithm was used for the phased-array imaging. This FUT array was attached on an Al alloy block of 30 mm thickness with a SDH of 3 mm diameter at the middle of block, as shown in the schematic of Figure 3(a). A high temperature gel couplant was used for this test. The longitudinal wave speed in Al alloy is 6,230 m/s. Because the span of the 16 elements is too long, only the first eight elements were utilized for imaging; the aperture of this array is 23 mm. The center of SDH was beneath and in between the 4th and 5th elements. The ultrasonic measurements using pulse-echo and pitch-catch modes on this block were performed at 150 °C. The center frequency of each element is 7 to 8 MHz. By multiplexing the transmitter, a matrix of A-scan signals *A_ij_*(*t*) were acquired for the *i*th transmitter and the *jth* receiver. Figure 5(a,b) show the pulse-echo signal *A*_44_(*t*–*t*_44_) and the pitch-catch signal *A*_45_(*t*–*t*_45_) of the target point (10 mm, 13.5 mm), which is the top of SDH.

**Figure 6.**
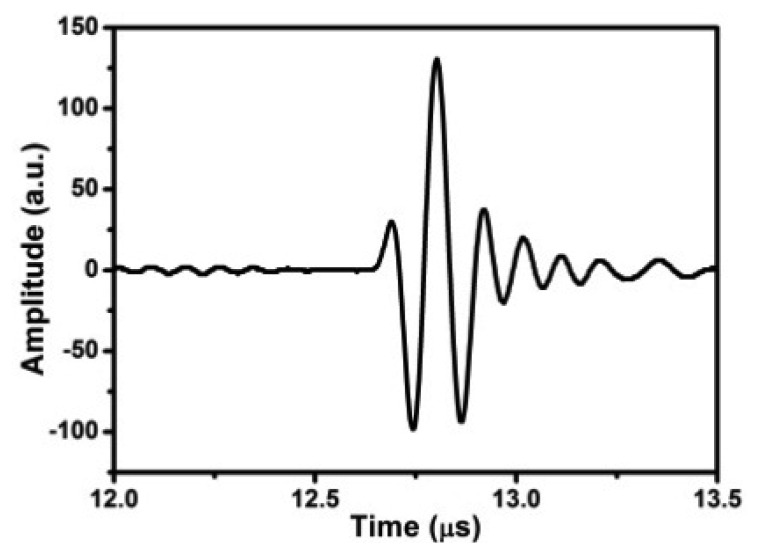
The pitch-catch signal *A*_12_ of the target point (0, 30 nm).

**Figure 7.**
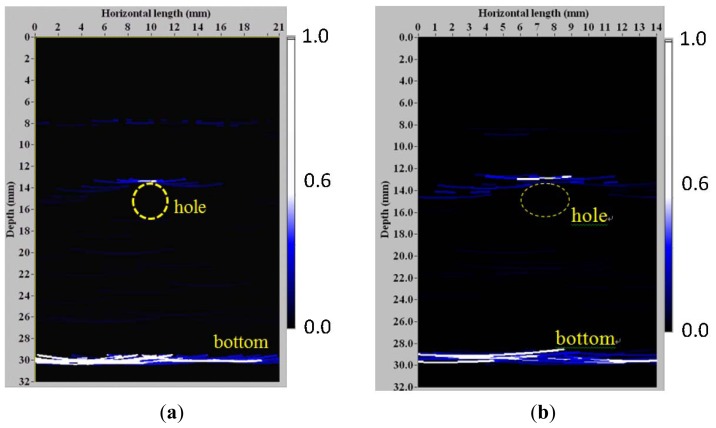
TFM images of (**a**) 8-elements FUT array (element size: 10 mm × 2 mm, gap: 1 mm) and (**b**) 7-elements FUT array (element size: 9 mm ×2 mm, gap: 0.5 mm) inspecting an Al alloy block of 30 mm thickness with a SDH of ϕ3 mm at the middle at 150 °C. Dashed circle: SDH.

Using Equation (1), a synthesized signal of *Sj*(**x**,*t*) of the *j*th receiver is reconstructed when multi-transmitters focus at the target point **x** in the block. The synthesized signal of the 4th receiver *S*_4_ for the target point (10 mm, 13.5 mm) is shown in Figure 5(c). In addition, the pitch-catch signal *A*_12_(*t*–*t*_12_) of the target point (0, 30 nm) at bottom is shown in Figure 6. Using the post-processing algorithm based on TFM, the phased-array imaging is reconstructed as shown in Figure 7(a), where the bottom of the back wall and the top of the SDH are indicated. The other smaller FUT array (element size: 9 mm × 2mm, gap: 0.5 mm) on a thinner Ti foil of 35 μm was also used to detect this SDH at 150 °C, where only the first seven elements were utilized for imaging; the aperture is 17 mm. The TFM image is shown in Figure 7(b). Again, the TFM phased-array image clearly shows the location of SDH and bottom of this Al alloy block. The two arrays of different apertures show the same performances for 3 mm SDH detection.

## 5. Conclusions

The applications of 1D FUT arrays, PZT-c film sprayed on Ti foils of 75 μm and 35 μm, to ultrasonic thickness measurement and defect detection for specimens at 150 °C were presented. We performed the thickness measurement on the curved surface of a steel pipe at 150 °C using FUT array to demonstrate the applicability on the in-service inspection for the internal corrosion detection of pipeline. The signal strengths obtained at 150 °C are about 7 dB weaker than those obtained at room temperature. Moreover, the FUT array was used to inspect a SDH in an Al alloy block. The ultrasonic measurements of the pulse-echo and pitch-catch modes using each single element as a transmitter and multi-elements as receivers of the FUT array were carried out. After that, a post-processing algorithm of TFM, which synthesizes the signals focusing at any target point in the inspected specimen, was implemented to process these pulse-echo and pitch-catch signals for obtaining the phased-array image. The phased-array measurement using FUT array for defect detection on a curved surface of steel pipe at 150 °C is still ongoing, and a miniaturized FUT array with a smaller element size and gap for the practical applications is being developed now to detect smaller defects. Moreover, the FUT arrays made from other sol-gels, e.g., bismuth titanate or lithium niobate, could be operable at higher temperatures, e.g., up to 400 °C [14,15].
